# Key Concepts to Identify the Role of Orthopedics in Child Development

**DOI:** 10.3390/children9071079

**Published:** 2022-07-20

**Authors:** Dai Sugimoto, Lyle J. Micheli

**Affiliations:** 1 Faculty of Sport Sciences, Waseda University, Tokyo 202-0021, Japan; 2 The Micheli Center for Sports Injury Prevention, Waltham, MA 02453, USA; lyle.micheli@childrens.harvard.edu; 3 Division of Sports Medicine, Boston Children’s Hospital, Boston, MA 02115, USA; 4 Department of Orthopedic Surgery, Harvard Medical School, Boston, MA 02115, USA

In order to examine the role of orthopedics in child development, longitudinal study designs are necessary. In the longitudinal design, it is ideal to have a follow-up period because it allows to track changes resulting from orthopedic intervention(s) over time. For instance, a group of shoulder patients with and without surgical intervention was followed for 5 years [1]. The main conclusion of this study was that, whether the patients had surgical intervention or not, their shoulders were better at the 5-year follow-up [1]. It is valuable to know that improvement of their shoulder conditions were noted at the 5-year mark. Needless to say, long-term follow-up is ideal in pediatric interventions to have a correct result of the medical or surgical intervention. Long-term follow-up studies of 10 or 15 years are efficient. 

Another major consideration is the type of population in the cohort. Who are we following over time? This is particularly important when children are a focus of the longitudinal study since they have many years of growth remaining. The growth can be expressed by various measures including chronological age, somatic age, and sexual age [2]. In the field of orthopedics, skeletal age is commonly used [3]. There are few orthopedic investigations which, meet the criteria of longitudinal effects of orthopedic interventions on skeletally immature children. 

In summary, it takes several critical components to identify the role of orthopedics in child development. We want to congratulate the studies published in this Special Issue. The evidence found in each investigation is invaluable from a scientific perspective. Next, these findings need to inform clinical practice, which should improve the health status of all children.
